# Interaction between Darunavir and Etravirine Is Partly Mediated by *CYP3A5* Polymorphism

**DOI:** 10.1371/journal.pone.0165631

**Published:** 2016-10-27

**Authors:** Leïla Belkhir, Laure Elens, Francis Zech, Nadtha Panin, Anne Vincent, Jean Cyr Yombi, Bernard Vandercam, Vincent Haufroid

**Affiliations:** 1 AIDS Reference Center, Department of Internal Medicine, Cliniques universitaires Saint-Luc, Université catholique de Louvain, Brussels, Belgium; 2 Louvain centre for Toxicology and Applied Pharmacology, Institut de recherche expérimentale et clinique, Université catholique de Louvain, Brussels, Belgium; 3 Integrated PharmacoMetrics, PharmacoGenomics and PharmacoKinetics, Louvain Drug Research Institute, Université catholique de Louvain, Brussels, Belgium; 4 Department of Clinical Chemistry, Cliniques universitaires Saint-Luc, Université catholique de Louvain, Brussels, Belgium; Azienda Ospedaliera Universitaria di Perugia, ITALY

## Abstract

**Objectives:**

To assess the impact of the loss-of-function CYP3A5*3 allele (rs776746, 6986A>G SNP) on darunavir (DRV) plasma concentrations.

**Methods:**

135 HIV-1 infected patients treated with DRV-based therapy were included in the study and plasma samples were obtained immediately before drug intake in order to determine DRV trough concentrations using an ultra performance liquid chromatography method (UPLC) with diode-array detection (DAD). Noteworthy is the fact that in 16 (11.9%) patients, etravirine (ETR) was combined with DRV. *CYP3A5* genotypes were determined using real time PCR method (TaqMan® genotyping assay). The patients were then classified into CYP3A5 expressors (CYP3A5*1 allele carriers) and non-expressors (CYP3A5*3 homozygous). Subsequently, the association between DRV plasma trough concentration ([DRV]_plasma_) and CYP3A5 genotype-based expression status was analyzed.

**Results:**

45% of the patients were classified as CYP3A5 expressors. In the whole cohort, mean [DRV]_plasma_ was not different between CYP3A5 expressors and non-expressors (1894ng/ml [CI95%: 1566–2290] versus 1737ng/ml [CI95%: 1468–2057], p = 0.43). However, in the subgroup of the 16 patients receiving DRV combined with ETR, a significantly lower [DRV]_plasma_ was observed for CYP3A5 expressors when compared to non-expressors (1385ng/ml [CI95%:886.3–2165] versus 3141ng/ml [CI95%:2042–4831], p = 0.007).

**Conclusions:**

Interaction between DRV and ETR is partly mediated by *CYP3A5* polymorphism with lower DRV plasma trough concentrations in CYP3A5 expressors suggesting a specific ETR-driven CYP3A5 activation only in CYP3A5 expressors. Consequently, these patients might be more at risk of infra-therapeutic [DRV]_plasma_. This potentially important observation is a good illustration of a genotype-based drug interaction, which could also have considerable consequences if translated to other CYP3A5-metabolized drugs. Further investigations are thus needed to confirm this association and to explore its clinical impact, mainly in the African population among whom CYP3A5 expressors are more frequent, before recommending systematic CYP3A5 pre-emptive genotyping for DRV-ETR co-administration.

## Introduction

Darunavir (Prezista® DRV) is the most recent potent protease inhibitor (PI) used as a component of highly active antiretroviral therapy (HAART) in combination with the pharmacokinetic (PK) booster ritonavir (association hereafter defined as DRV/r) for the treatment of Human Immunodeficiency Virus-1 (HIV-1) infected patients [1, 2]. DRV has demonstrated potent *in vitro* and *in vivo* activity against both wild-type (WT) and PI-resistant HIV [3–5].

Initially, DRV/r regimen was approved at a dose of 600mg/100mg twice daily for treatment-experienced HIV-infected patients on the basis of clinical efficacy and safety data collected in the POWER 1 and 2 trials [3, 6, 7].

Later, in the ARTEMIS trial, the efficacy of a DRV/r once daily regimen (800/100 mg) was demonstrated in treatment-naïve patients. The study revealed that DRV/r QD was not only non-inferior but also, significantly superior in terms of virologic response when compared to lopinavir/ritonavir after 96 weeks in antiretroviral-naive patients [8]. Consequently, DRV was approved at two different daily dosages depending on the clinical setting and the genotypic testing of the virus.

As many other PI, DRV is a substrate and inhibitor of CYP3A enzymes and is almost exclusively metabolized by these CYP3A isoforms [4, 6, 9]. DRV is also a substrate of the efflux transporter P-glycoprotein (P-gp) [6]. Ritonavir (RTV) is a potent inhibitor of CYP3A that reduces DRV clearance, resulting in a 14-fold increase of DRV exposure and extends DRV half-life up to 15 hours [10].

Etravirine (Intelence® ETR) is a second-generation non-nucleoside reverse transcriptase inhibitor (NNRTI) that possesses an activity against both WT and NNRTI-resistant HIV [11, 12] and a high genetic barrier against the development of drug resistance. Two studies (DUET-1 and DUET-2) [13, 14] conducted in clinically advanced, treatment-experienced patients with viral strains harboring NNRTI and PI resistances have shown superiority of ETR to the placebo in terms of virological efficacy, immunological recovery and clinical progression. Noteworthy in this difficult-to-treat population, ETR was associated with an optimized background regimen (OBR) that included DRV/r 600/100 mg BID.

ETR is primarily metabolized in the liver via CYP2C19, CYP2C9, and CYP3A [15]. ETR is also a weak inducer of CYP3A4 [16] (by increasing the abundance of CYP3A4 mRNA in a pregnane X receptor (PXR) dependent manner) [17] and an inhibitor of CYP2C9, CYP2C19 and P-gp [15, 16, 18]. It has been previously demonstrated that the *CYP3A5* gene is inducible by mechanisms similar to those involved in *CYP3A4* induction, involving constitutively activated receptor (CAR) and PXR [19]. Therefore, as an established CYP3A4 inducer [16], ETR could also be considered as a potential CYP3A5 inducer but only in individuals expressing this isoform (see below). The CYP3A induction and P-gp inhibition driven by ETR has the potential to alter DRV disposition and therefore, theoretically, there exists a potential hazard of drug-drug interactions between these agents.

The CYP3A proteins (mainly CYP3A4 followed by CYP3A5) are the most abundant cytochrome P450 (CYP) proteins in human liver and small intestine and they metabolize around 50% of drugs currently in use [20]. While functional genetic polymorphisms for *CYP3A4* genes are rare [21], CYP3A5 activity highly depends on genetic status of the patient [22].

The most common loss-of-function variant of *CYP3A5* is designated as CYP3A5*3 (rs776746, 6986A > G) [22, 23]. This single nucleotide polymorphism (SNP) consisting of a substitution within intron 3 creates a cryptic splice site, affecting mRNA splicing and resulting in a premature stop codon that finally gives rise to the translation of a truncated nonfunctional protein [22, 23]. Consequently, only carriers of at least one CYP3A5*1 (wild-type) allele are expressing a functional CYP3A5 enzyme whereas individuals homozygous for the loss-of-function allele (CYP3A5*3/*3) are considered to be CYP3A5 non-expressors.

Furthermore, distribution of *CYP3A5* polymorphisms varies greatly across the globe with the CYP3A5*3 allele occurring with different frequencies among ethnic populations. The CYP3A5*3 allele is abundantly present in Caucasian population but rare in African population (allelic frequency of 91–95% [23, 24] and 27% [23], respectively). Therefore, CYP3A5 expressors are mainly encountered in the population of African origin.

In Western and Central Europe, in 2014, 37% of all new HIV infections occurred among migrants from outside of this region[25]. For example, in the UK in 2014, 55% of men and 62% of women living with HIV were from sub-Saharan Africa; in Belgium, when the data was available, almost 30% of the newly infected HIV people were of African origin [26]. In the USA in 2013, 46% (506000) of people living with HIV were African Americans, representing on the whole, 12% of the US population [27].

Considering that CYP3A5 may represent up to 50% of the total hepatic CYP3A content in CYP3A5*1 allele carriers [22], the *CYP3A5* genetic polymorphism may be therefore the most important genetic contributor not only to interindividual but also to interracial differences in CYP3A-dependent drug clearance.

Therefore, the aim of this study was to assess the impact of the loss-of-function CYP3A5*3 allele on DRV plasma concentrations in HIV-1 infected patients co-treated or not with ETR.

## Materials and Methods

This study was conducted at the AIDS Reference center of Cliniques universitaires Saint-Luc in Brussels, Belgium. HIV-1 infected patients of 18 years and older treated by DRV/r for at least one month prior to inclusion were eligible for the study and recruited between November 2012 and June 2015. The study protocol (NCT02514369) was approved by the Ethical Committee of UCL Saint-Luc: "Comité d'Ethique hospitalo-facultaire" (National number: B403; approval: B403201214460). All patients included provided their written informed consent to participate to the study. In total, 149 HIV-1 infected patients were recruited.

After a clinical assessment and in addition to the samples routinely collected for the clinical follow-up (viral load, CD4 cell count), two more blood samples were drawn immediately before drug intake, with the highest timing precision conceivable given the ambulatory context of the study recruitment. These samples were used for determination of DRV plasma through concentration ([DRV]_plasma_) and for genomic DNA isolation, respectively. In order to obtain a post-intake delay as close as possible to the trough sampling time, each patient was personally contacted by phone a few days before the study visit to ensure not taking the medication prior to blood sampling.

To quantify [DRV]_plasma_, blood samples were obtained on heparinized tubes and immediately centrifuged at 1125x*g* for 10min at room temperature (RT). Plasma was then collected and stored at -20°C until the day of quantification.

The [DRV]_plasma_ was measured using an ultra performance liquid chromatography (UPLC) method with diode array detection (DAD)[28] based on a method routinely used in our laboratory. Using this validated method, the laboratory has obtained successful results in the external quality control program organized by SKML (The Netherlands) on antiretroviral drugs, including DRV.

The second blood sample was drawn in an EDTA tube and stored at -20°C until the day of genotyping analysis. Genomic DNA was extracted from whole blood using a QIAamp® DNA Mini Kit ^TM^ (Qiagen, CA, USA). Allelic discrimination for the determination of *CYP3A5* 6986A > G was performed using real time PCR TaqMan® (Applied Biosystems, CA, USA) genotyping assay (C_26201809_30) on the StepOnePlus ^TM^ Real Time PCR System (Applied biosystems, CA, USA).

[DRV]_plasma_ were log-transformed for normalization of the distribution before further analysis. They were then reported as geometric mean of plasmatic DRV concentration (mean [DRV]_plasma_). Genotype and allele frequencies were calculated and deviations from Hardy-Weinberg equilibrium (HWE) were evaluated using exact tests.

The patients were also classified into CYP3A5 expressors (CYP3A5*1 allele carriers) and CYP3A5 non-expressors (CYP3A5*3 homozygous).

Subsequently, the associations between [DRV]_plasma_ and CYP3A5 genotype-based expression status were analyzed both in the entire cohort and in the subgroup of patients with or without co-administration DRV-ETR.

Statistical analyses were performed using JMP Pro 12 version 12.0.1 for MAC (SAS Institute Inc., Cary, NC, USA).

The comparison of the [DRV]_plasma_ with the different CYP3A5 groups was performed using the two-tailed Mann-Whitney *U* test. A p-value < 0.05 was deemed statistically significant.

## Results

Fourteen out of the 149 patients were excluded because of suspected non-compliance (n = 6) or non-classical DRV dosage (DRV 600mg QD, DRV 900mg QD, DRV 1200mg QD) (n = 7), while one patient was excluded because of substantial deviation from the drug intake protocol (DRV intake just before blood sampling). The clinical characteristics of the 135 remaining patients are reported in Table 1.

**Table 1:** Patients characteristics at day of sampling.

Number of patients included	135
DRV dosage repartition, n (%)	
• 800mg once daily • 600mg twice daily	• 93 (69%) • 42 (31%)
Co-administered ARV drugs	
• NNRTI	ETR (n = 16) NVP (n = 2) NNRTI-free (n = 117)
• NRTI	ABC/3TC (n = 21),FTC/TDF (= 67), 3TC (n = 16), TDF245mg (n = 8), NRTI-free (n = 25)
• INSTI	RAL (n = 38), DTG (n = 4)
• MVC	n = 13
Age, years (mean ± SD)	48 ± 12.4
Body Mass Index, kg/m^2^ (mean ± SD)	25.4 ± 4.9
Gender , n (%), male	86 (64%)
Ethnic origin, n (%)	
• Caucasian	85 63%)
• African	49 36%)
• South American	1 (1%)
CD4 cell count, cells per μl (median [min-max])	532 [33–1641]
Nadir CD4 cell count, cells per μl (median [min-max])	149 [2–681]
HIV-1 RNA < 37 copies per mL, n (%)	103 6%)
• HIV-1 RNA > 37 copies per mL, n(%) • copies per mL (median [min-max])	• 32 (24%) • 96 [37–1687]
Duration of treatment, months (mean ± SD)	24.9 ± 22.8
Post-intake delay, hours (mean [CI95%])	
• DRV/r 800/100mg once daily	20.0 [18.7–21.4]
• DRV/r 600/100mg twice daily	14,5 [13.5–15.1]
eGFR, mL/min/1,73m^2^ (mean ± SD)	79.6 ± 16.2

DRV: Darunavir, ARV: antiretroviral, NNRTI: Non-Nucleoside Reverse Transcriptase Inhibitor, NRTI: Nucleoside Reverse Transcriptase Inhibitor, INSTI: Integrase Strand Transfer Inhibitor, RAL: raltegravir, DTG: dolutegravir, MVC: maraviroc ETR: etravirine, NVP: nevirapine, ABC: abacavir, 3TC: lamivudine, FTC: emtricitabine, TDF: tenofovir disoproxil fumarate, NNRTI-free: without any NNRTI, NRTI-free: without any NRTI, eGFR: estimated glomerular filtration rate by utilizing the Modification of Diet in Renal Disease (MDRD) study equation

Sixteen out of the 135 included patients (86 (64%) male, mean age 48 ± 12.4 years) received a regimen comprising DRV/r and ETR (200mg BID) in co-administration with 13 of these patients receiving DRV/r at the 600/100mg twice daily regimen.

In terms of ethnic origin, 85 (63%) patients were Caucasian, 49 (36%) were African and one patient originated from South-America.

The mean duration of the treatment was 24.9 ± 22.8 months. 103 (76%) patients had an undetectable viral load (< 37 cps/ml) and among the remaining 32 patients, the median plasma HIV-RNA level was 96 cps/ml [min-max: 37–1687].

Among the 135 patients, 45% were classified as CYPA5 expressors (CYP3A5*1/*1 and *1/*3) (Table 2). Considering the subgroup of the 16 patients receiving DRV combined with ETR, 50% were CYPA5 expressors. The CYP3A5*1 allele frequencies were 77.6% and 9.4% in African and Caucasian patients, respectively. The *CYP3A5* genotype distributions were conformed to Hardy-Weinberg equilibrium.

**Table 2:** Distribution of the *CYP3A5* genotypes in the study cohort and according to the ethnicity (Caucasian versus African origin).

		Study Cohort	Caucasian	African
CYP3A5*3/*3	CYP3A5 non expressors	75 (55%)	70 (82.5%)	4 (8%)
CYP3A5*1/*3	CYP3A5 expressors	28 (21%)	14 (16.5%)	14 (29%)
CYP3A5*1/*1		32 (24%)	1 (1%)	31 (63%)
Total		135	85	49

Overall, mean [DRV]_plasma_ was 1805ng/ml [CI95%: 1593–2045]. There was no statistically significant correlation between age, gender, ethnicity, weight and [DRV]_plasma._

When considering the entire cohort, CYP3A5 allelic status did not significantly influence mean [DRV]_plasma_ with values of 1894ng/ml [CI95%: 1566–2290] and 1737ng/ml [CI95%: 1468–2057] for CYP3A5 expressors and CYP3A5 non-expressors, respectively (p = 0.43) (Table 3).

**Table 3:** DRV plasma concentration according to CYP3A5*3 polymorphism.

	CYP3A5 non-expressors	CYP3A5 expressors	P-value
• Study cohort • n = 135	• 1737ng/ml • (CI95%: 1468–2057) • n = 75	• 1894ng/ml • (CI95%:1566–2290) • n = 60	0.43
• Subgroup DRV/r 800/100mg QD • n = 93	• 1498ng/ml • (CI95%: 1227–1828) • n = 52	• 1693ng/ml • (CI95%:1314–2182) • n = 41	0.34
• Subgroup DRV/r 600/100mg BID • n = 42	• 2430 • (CI95%: 1817–3249) • n = 23	• 2411 • (CI95%: 1895–3067) • n = 19	0.92
• Patients without ETR • n = 119	• 1619ng/ml • (CI95%: 1356–1932) • n = 67	• 1987ng/ml • (CI95%: 1611–2451) • n = 52	0.07
• Subgroup DRV/r plus ETR • n = 16	• 3141ng/ml • (CI95%: 2042–4831) • n = 8	• 1385ng/ml • (CI95%:886.3–2165) • n = 8	0.007
• Subgroup DRV/r 600/100mg BID plus ETR • n = 13	• 3141ng/ml • (CI95%: 2042–4831) • n = 8	• 1486ng/ml • (CI95%:725–3044) • n = 5	0.034

DRV plasma concentrations are expressed as geometric mean and geometric 95% confidence interval (CI95%)

**Fig 1:**
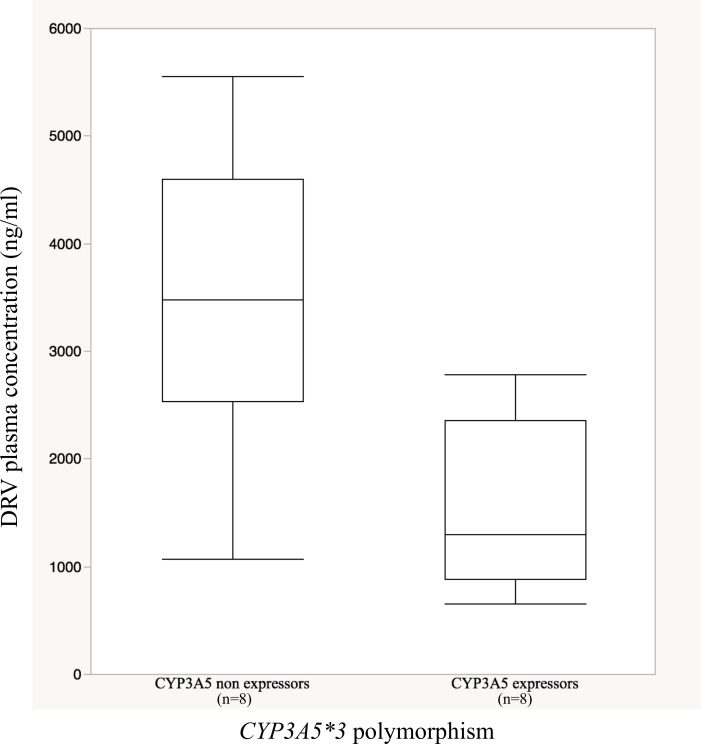
Darunavir plasma concentration according to CYP3A5*3 polymorphism in patients co-treated with darunavir and etravirine. Values are reported on the Y-axis using a box and whisker plot. Bottom and top of the boxes indicate the 25th and 75th percentiles, respectively and the inside-line represents the 50th percentile (median). Whiskers show maximal and minimal observed values.

However, when analyzing the data for the 16 patients receiving ETR in co-administration with DRV, significantly lower mean [DRV]_plasma_ were observed for CYP3A5 expressors when compared to non-expressors (1385ng/ml [CI95%:886.3–2165] versus 3141ng/ml [CI95%:2042–4831], p = 0.007) (Fig 1 and Table 3). This difference was also significant in the subgroup of the 13 patients receiving exactly the same DRV and ETR regimen.

For the 119 patients without ETR co-administration, CYP3A5 allelic status did not significantly influence mean [DRV]_plasma_ with values of 1987ng/ml [CI95%: 1611–2451] and 1619ng/ml [CI95%: 1356–1932] for CYP3A5 expressors and CYP3A5 non-expressors, respectively (p = 0.07). (Table 3).

## Discussion

In our study, we showed that [DRV]_plasma_ was significantly lower in the CYP3A5 expressor than in the non-expressor group when ETR is co-administered suggesting a specific ETR-driven CYP3A5 induction but only in CYP3A5 expressors.

Drug-drug interactions between antiretroviral drugs are complex and this aspect remains a challenge for most clinicians.

As ETR is an inducer of CYP3A, and an inhibitor of CYP2C9, CYP2C19 and P-gp, we could expect that its co-administration with drugs that are substrates of these drug-metabolizing enzymes and/or drug transporters may alter their PK profile. Indeed, drug-drug interactions have been demonstrated between ETR and other antiretroviral agents that are substrates of CYP3A. As an example, Kakuda et al. demonstrated that co-administration of ETR with maraviroc in 14 healthy predominantly Caucasian (93%) adults, decreased maraviroc exposure by 53% (AUC_0–12h_). As maraviroc is mainly eliminated following CYP3A4 biotransformation, this observation would suggest a significant CYP3A induction mediated by ETR [29]. Interactions with ETR have also been described with PI such as atazanavir (ATV), which is also a CYP3A substrate. Indeed, it was shown in an open-label crossover study conducted in 32 healthy volunteers that when ETR was co-administrated with unboosted ATV (400mg QD, n = 16, 38% Caucasian, 19% Black, 44% Hispanic) or with boosted ATV/r (300/100mg QD, n = 16, 63% Caucasian, 19% Black, 19% Hispanic), ATV trough concentration was decreased by 38 and 47% with or without ritonavir, respectively [30]. The effects observed in the latter two studies could be due to an induction of CYP3A enzymes by ETR.

We have previously demonstrated a possible interaction between DRV and ETR with ETR co-administration decreasing [DRV]_plasma_ by approximately 50% [31]. It was the first time that such an interaction was described between both drugs and this data suggested a potential induction of CYP3A isoforms by ETR.

The conclusion of the absence of major interaction between DRV and ETR, as mentioned in the prescribing information of DRV[6] or ETR[15], is mainly based on pharmacokinetic substudies of the phase 3 trials DUET-1 and DUET-2 whereas DRV/r 600/100 mg bid was included in the background regimen of patients with ETR. Noteworthy is the fact that the majority of the patients receiving ETR in these phase 3 trials were of Caucasian origin (65% and 77% in DUET-1 and DUET-2, respectively) with an expected low prevalence of CYP3A5 expressors while only few of them were of Black origin (14% and 13% in DUET-1 and DUET-2, respectively)[13, 14]. Four other studies (as detailed below) [32–35] concluded that no dose adjustments were required when DRV and ETR were co-administered.

In the phase I, open-label, randomized, two-way crossover study of Schöller-Gyüre et al involving 32 healthy volunteers, it was observed that co-administration of DRV/r (600/100mg bid) with ETR (100mg or 200mg bid) resulted in unchanged DRV PK parameters (in particular similar C_trough_ and C_max_) with the exception of a slight 15% increase in mean DRV AUC_0–12h_ when combined with ETR 200mg bid compared to DRV alone [32]. Conversely, co-administration of DRV/r with ETR 100mg twice daily decreased ETR exposure by 37% when compared to ETR alone, which might reflect the inductive effect of CYP2C9 and CYP2C19 isoforms by ritonavir [36]. However, in this cohort of Schöller-Gyüre et al, 94% of the patients were of Caucasian origin, and no African volunteers were included. The situation was clearly different in our cohort, where 36% of the patients were of African origin with a higher probability of CYP3A5 expression, as explained above. In addition, in their study, out of the 32 volunteers, only 23 completed the trial, which greatly reduced the power of the statistical analysis (13 and 10 in the group ETR 100mg bid and 200mg bid, respectively).

The study of Boffito et al. included 10 HIV-1 infected patients receiving DRV/r (600/100mg bid) combined with ETR (200mg bid) that were mostly Caucasians (7 out of 10)[33]. In that particular study, the authors demonstrated that DRV exposure was similar to “historical control” (obtained from subjects taking either ETR without DRV or DRV in the absence of ETR) [33].

Later, Barrail-Tran et al. studied the interaction between DRV/r (600/100mg bid) or raltegravir (RAL, 400mg bid) and ETR (200mg bid) on a small group of 10 HIV-1 infected patients with previous multiple treatment failure [34]. Surprisingly, the addition of ETR leaded to an increase of DRV C_trough_ and AUC by 71% (from 2241ng/ml to 3837ng/ml) and 14%, respectively. Unfortunately, no data was available concerning the ethnic origin of the patients. All these patients were heavily pretreated prior to enrollment with other ARV drugs including drug-metabolizing enzymes and/or transporter inhibitors or inducers. Therefore it remains unknown if the increase in DRV exposure after the addition of ETR in this study was due to ETR or to the effect of the concomitant ARV drugs.

The only cohort involving a large proportion of Black people was conducted by Kakuda et al. [35]. This multicenter study included 376 patients among whom 190 co-treated with DRV/r (600mg/100mg bid) and ETR (200mg bid) (60% Black, 22% Hispanic, 17% white and 1% Asian or other). They demonstrated that pharmacokinetic exposure to DRV was not influenced by race, sex, age, body weight or use of ETR or TDV but, as pinpointed by the authors themselves, this study was “not specifically powered to compare the effects of these covariates on DRV PK”.

In our study, we have highlighted a significant impact of the *CYP3A5* polymorphism on the PK interaction between DRV and ETR. In contrast to our results, the majority of the above described studies concluded to no clinically relevant interaction between DRV and ETR. However, all these studies were performed either predominantly on subjects of Caucasian origin with only a small proportion of African or Afro-American subjects [32–34] or without stratification allowing to test the effect of ethnicity/CYP3A5 expression in the sub-group of patients co-treated by DRV and ETR [32–35]. The frequency of CYP3A5*1 carriers (CYP3A5 expressors) is indeed very low in Caucasians contrasting with the high frequency reported among Africans who are predominantly CYP3A5 expressors (73%). When expressed, CYP3A5 can represent more than 50% of the total hepatic CYP3A content[22]. With this in mind, it might be one of the most important genetic contributors to interindividual and interracial differences in CYP3A-dependent drug metabolism.

In the 16 patients in our study treated by DRV combined with ETR, 6 were of African origin (38%), and all were classified as CYP3A5 expressors based on their genotype. This frequency is presumably much higher than in previous cited studies [13, 14, 32, 33]. The 10 other ETR/DVR/r patients were of Caucasian origin, including 8 CYP3A5 non-expressors. Given that in CYP3A5 expressors, CYP3A5 is the predominant CYP3A isoenzyme, one can hypothesize that, in this particular population, CYP3A5 influences the DRV metabolism maybe more than other CYP3A isoforms, i.e. CYP3A4. The fact that no difference was observed in [DRV]_plasma_ between CYP3A5 expressors and non-expressors in the overall study population (n = 135) or in the subgroup receiving only DRV/r without ETR (n = 119) could be partly explained by the CYP3A inhibition by RTV. Such an effect has been previously reported for saquinavir (SQV) with significant difference observed between *CYP3A5* genotype with the unboosted regimen on one hand [37] and no difference with the boosted SQV/r regimen[38]. Therefore, the CYP3A induction by ETR could partly compensate its inhibition by RTV making the effect of *CYP3A5* genotype visible. Although not supported by our experimental data, a similar effect could be theoretically expected in patients treated with cobicistat, a more recent DRV booster.

In the sub-group of patients co-treated with DRV/r and ETR, the mean [DRV]_plasma_ was 1385ng/ml [IC95%:930.7–2062] for CYP3A5 expressors. This value is below the recommended consensus for treatment-experienced patients which is between 1800 and 2000 ng/ml[39]. Surprisingly, neither the prescribing information of DRV or ETR [6, 15] nor the consensus website www.hiv-druginteractions.org (University of Liverpool) mention any caution when DRV is combined with ETR particularly in patients more difficult to treat with multidrug resistant virus and limited therapeutic options.

Among these 16 patients receiving DRV/r and ETR, only one had a detectable viral load (110 copies/mL at day of inclusion, undetectable 6 months after but with 3 blips during the 2 years of follow-up). This particular case was a woman of African origin, CYP3A5 expressor with a low [DRV]_plasma_ at 652 ng/ml.

This potentially reflects a modest clinical consequence of this low DRV exposure in CYP3A5 expressors but it is important to stress that all these 16 patients were heavily treated and maybe then “protected” against a virological rebound by the other associated ARV drugs (robust backbone). Nonetheless, sub-optimal drug exposure increases the likelihood of viral rebound resistance and may confer resistance to the drugs in the current regimen and/or cross-resistance to other drugs of the same class by decrease drug pressure. So, knowing that a drug-drug interaction in a determined population (CYP3A5 expressors) may decrease a plasma drug concentration by more than 55% remains useful information, in our opinion, particularly in patients with limited options treatment and/or with suboptimal backbone.

The present study has however some limitations. First, our cohort of patients co-treated with DRV and ETR is still quite small, although bigger than most of previous published studies. Then, this drug-drug interaction mediated by a genetic polymorphism should be studied in a larger cohort to evaluate its potential clinical impact.

Secondly, the dosage of the C_trough_ as unique DRV PK parameter probably does not reflect the daily drug exposure as best as full AUC determination. However, as this (preliminary) study was performed in real life with ambulatory patients, the C_trough_ was the only parameter that we could obtain. Furthermore, C_trough_ represents the only parameter useful and available in daily practice. Moreover, therapeutic drug monitoring (TDM) based on drug trough plasma concentrations has shown its efficacy and has been successfully applied in clinical settings to optimize HIV treatment management by improving efficacy and reducing toxicity in particular scenarios such as pregnancy with risk factors for virologic failure, and drug-drug interaction [40]. So, this one-time point measurement remains a suitable parameter to evaluate DRV exposure and reflects quite rationally real life in clinical practice settings.

## Conclusions

ETR seems to boost DRV elimination but only significantly among CYP3A5 expressors, suggesting a specific CYP3A5 activation. Our study has highlighted that CYP3A5 expressors might be more at risk of infra-therapeutic DRV plasma concentrations when ETR is included in their therapeutic regimen. Knowing that the majority of people of African origin are CYP3A5 expressors, this observation may be clinically relevant in this population at risk of being under the therapeutic range, especially in patients with limited drug options and/or unable to take integrase strand transfer inhibitor (INSTI).

This result is a good illustration of a genotype-based drug interaction that could have also considerable consequences if translated to other CYP3A5-metabolized drugs. More generally, this information could help physicians to understand the relationship between ethnic origin and predisposition to drug response in order to improve therapies.

Further investigations are thus needed to confirm this association and to explore its clinical impact before recommending systematic *CYP3A5* pre-emptive genotyping. We believe it may be wise to modify the current prescribing information of DRV and ETR [6, 15] and/or the consensus website www.hiv-druginteractions.org (University of Liverpool) to advise caution when DRV is combined with ETR in this particular setting.
